# Changes in head staff members in male elite-level football teams are associated with increased hamstring injury burden for that season: the UEFA Elite Club Injury Study

**DOI:** 10.1136/bmjsem-2023-001640

**Published:** 2023-11-15

**Authors:** Jan Ekstrand, Wart Van Zoest, Håkan Gauffin

**Affiliations:** 1 Department of Health, Medicine and Caring Sciences, Linköping University, Linkoping, Sweden; 2 Orthopaedic Surgery, St. Anna Hospital, Geldrop, The Netherlands; 3 PSV, Eindhoven, The Netherlands; 4 Department of Orthopaedics Department of Biomedical and Clinical Sciences, Linköping University, Linkoping, Sweden

**Keywords:** Fatigue, Muscle damage/injuries, Soccer, Sporting organisation

## Abstract

**Objective:**

To evaluate whether a change of head coach or other head staff before or during a season is correlated to hamstring injury (HI) burden in male elite-level football (soccer) in Europe.

**Methods:**

The survey was conducted using a questionnaire reporting any staff change within the team. Data about the head staff changes and hamstring injury burdens were collected from 14 teams participating in the Elite Club Injury Study (ECIS) during the 2019/2020, 2020/2021 and 2021/2022 seasons.

**Results:**

On average, replacing the head coach before or during a season happens in every second season. All changes, except for the change of the head coach during a season, indicate an association with an increase in HI burden (ranging from 10% to 81%). However, only changes in the fitness coach and team doctor roles reached statistical significance. The HI burden seems to be influenced by adding new staff members, such as the head of fitness/performance coach in 36% of the teams and the team doctor in 17%. New head coaches starting the season with their own, for the team new, fitness/performance coach was highly associated with increased HI burden (p<0.001).

**Conclusions:**

Bringing their own fitness/performance coaches is common for managers entering a new elite male football club. However, this paper has highlighted that this trend seems to lead to a three times increase in HI burden. Similarly, replacing the team doctor was also associated with increased HI burden. Instability among head staff members in male elite-level football teams seems associated with increased HI burden during the season.

**WHAT IS ALREADY KNOWN ON THIS TOPIC**

- Coaching replacement is a frequent phenomenon in elite-level football, and its impact on a team’s success is debatable.
- Hamstring injury (HI) is the most common injury in professional football (soccer).
- Recent research has indicated that coach replacement might increase the number of muscle injuries within teams.

**WHAT THIS STUDY ADDS**

- The head coach replacement before or during a season was the most common change among head staff members; it took place in every second team season.
- Replacements of the head of fitness/performance coach and the team doctor were associated with increased HI burden, but an isolated head coach replacement was not.
- The most distinct increase in HI burden was noticed when a new coach simultaneously brought his own new fitness/performance coach to the team.

**HOW THIS STUDY MIGHT AFFECT RESEARCH, PRACTICE OR POLICY**

- Elite football clubs must develop and invest in their performance staff to create continuity within their department.
- The transition to a new fitness/performance coach or team doctor should be completed with the utmost care and accompanied by a thorough and precise handover with the former staff.

## Introduction

Hamstring injury (HI) is the most common injury subtype in male professional football players.^1–3^ During the recent eight seasons (2014/2015 to 2021/2022), the incidence and burden of HIs during training and match play have increased significantly in male elite-level football in Europe,^2^ and the proportion of injuries diagnosed as HIs increased from 12% in 2001/2002 to 24% in 2021/2022.^2^

Various risk factors for HIs have been proposed.^4–8^ According to the CMOs (Chief Medical Officers) of male elite-level football in Europe, most risk factors are associated with variables that coaches can influence. As a result, coaches influence the injury situation within elite clubs.^7^

A recent study from the Turkish Super League indicated that replacement of coaches might be a risk factor and increase the number of muscle injuries in teams.^9^ However, this study had limitations, such as a small sample size, as it only tracked two teams throughout three seasons.

We wanted to evaluate the effect of staff replacements on HI burden in a larger sample size of 14 Elite Club Injury Study (ECIS) teams followed for 3 seasons.

The objective of this study was to evaluate how often head coaches were replaced before or during a season and whether such replacements are correlated to the HI burden in male elite-level football in Europe. A second aim was to examine whether HI burden was associated with replacing other staff members, including the head of performance/fitness coach, head of the medical department or head of physiotherapy.

It was hypothesised that the stability of the team behind the team is important, and replacement of the coaches, performance or medical staff before or during a season would be common and negatively impact the HI burden for that season.

## Material and methods

### Study design

In 1999, the Union of European Football Associations (UEFA) initiated a research project to reduce injuries and increase player safety in male professional football—the ECIS.^1 2 10–12^ This current study was an observational cohort study with prospectively collected injury data and retrospectively collected data investigating changes in the team’s staff in clubs participating in the ECIS.

### Study participants

A total of 14 clubs participated in ECIS and qualified for the initial stage of either the UEFA Champions League(UCL) or Europa League (EL) in the three latest seasons (2019/2020, 2020/2021 and 2021/2022). These 14 teams delivered complete injury data for all three seasons, and all teams answered a questionnaire (response rate 14/14=100%). The 14 teams represented 8 countries (3 from England, 3 from Germany, 2 from the Netherlands, 2 from Spain and 1 from France, Portugal, Belgium and Italy). All the 14 teams followed the traditional Western European season, with a preseason training period starting in July and a competitive league’s season starting in August and ending in May. The preseason training period was defined as the period from the first training session to the first competitive match (in the national league or the European Cups, for instance, qualification for the group stage (UCL or EL)).

### Exposure and injury data collection

The three seasons’ exposure and injury data collection was completed in October 2022. Injuries not resolved yet were followed until March 2023, when the return to play was completed. Definitions and data collection methods in ECIS have been described in detail previously.^13–15^ In brief, teams assigned a contact person (a medical staff member) responsible for registering data. Contact persons were given a manual that provided study methods and operational definitions used in the study. Injury was defined as ‘Any physical complaint sustained by a player resulting from a football match or football training that leads to the player being unable to take part in football training or match play thereafter fully’. A hamstring injury was ‘A traumatic distraction or gradual onset injury to the hamstring muscle group’. HI burden is expressed as number of days lost per 1000 hours of exposure.

Teams were requested to provide the study group with monthly exposure and injury data. Members of the study group reviewed all data to ensure that it complied with the study protocol. If any missing or unclear data were identified during this review, immediate feedback was sent to the contact person to complete or correct the data.

### The survey questionnaire

A questionnaire investigating changes in the team’s staff was the base of the survey. The questionnaire covered three seasons: 2019/2020, 2020/2021 and 2021/2022 (online supplemental box 1). It included questions about the change of four main practitioners within the team: (1) head coach, (2) head of performance/fitness, (3) head team doctor and (4) head of physiotherapy.

10.1136/bmjsem-2023-001640.supp1 Supplementary data

The questionnaire was sent to the CMOs of each club. After agreeing to participate in the study, they were provided access to the questionnaire using the online survey software SurveyMonkey (SurveyMonkey, California, USA). To increase validity and avoid recall bias, the questionnaire data was crosschecked with data about head-coach changes at Transfermarkt (https://www.transfermarkt.com/).

The questionnaire was distributed in February 2023. The survey software distributed automatic reminders after 3, 7 and 10 days. All 14 teams completed the questionnaire within 1 month.

### Missing data

One club did not provide an answer to Question 24 (Was the performance/fitness coach of the 2021/2022 season brought in by the head coach or by the club?).

### Patient and public involvement

This research was carried out without patient (player) involvement, that is, players were not invited to comment on the study design or contribute to drafting this document.

### Equity, diversity and inclusion

This study was conducted on male professional football players only. A women’s ECIS was launched in July 2017 in collaboration with the UEFA with similar data collection on HIs. Results from this study will be presented in the future.

### Data analyses

The HI burden was used as an outcome measure. Injury burden was calculated as the number of lay-off days per 1000 hours of exposure. Teams with changes of head staff members (head coach, head fitness/performance coach, main responsible team doctor or physiotherapist) were compared with teams without these changes. The Student’s t-test was used for between-group analysis, including CIs. Analyses were two-sided, and the significance level was p<0.05.

## Results

All 14 CMOs replied to the survey. The frequency of head staff replacement and their associations with HI burden is shown in table 1. In total, 49 changes of head staff took place in 25 out of the 42 seasons (60%). Six teams made 5–9 replacements during the 3 seasons, 5 teams between 1–4, while 3 teams did not replace any of the four head positions during the study time. The most frequent change was replacing the head coach (manager). Introducing a new head coach was seen in 20 of the 25 (80%) team seasons with changes of head staff.

**Table 1:** Replacement of staff and the hamstring injury (HI) burden for team seasons with replacements compared with team seasons without replacements

Replacement of staff	Frequency of replacements	HI burden for team seasons without replacements (mean, 95% CI)	HI burden for team seasons with replacements (mean, 95% CI)	HI burden change when replacements compared without replacements	HI burden increase when team season replacement compared with without replacement (95% CI)
Total number of replacements during the 42 team seasons	49	24 (15 to 34)	32 (23 to 40)	+33%	8 (−6 to 21)
New head coach at the start of the season	10/42=24%	27 (20 to 31)	35 (24 to 46)	+30%	8 (−7 to 23)
Change of head coach during the season	10/42=24%	29 (20 to 37)	29 (12 to 45)	±0	0 (−16 to 14)
New head coach before or during the season	20/42=48%	27 (19 to 35)	32 (22 to 42)	+19%	5 (−8 to 18)
New head fitness/performance coach at the start of the season	10/42=24%	27 (19 to 34)	38 (28 to 47)	+41%	11 (−4 to 26)
Change of head fitness/performance coach during the season	7/42=17%	27 (20 to 33)	40 (20 to 60)	+48%	3 (−3 to 30)
New head fitness/performance coach before or during the season	15/42=36%	24 (17 to 31)	39 (28 to 50)	+63%	15 (3 to 28)
New head team doctor before or during the season	7/42=17%	26 (20 to 31)	47 (23 to 70)	+81%	21 (5 to 37)
New head physiotherapist before or during the season	7/42=17%	29 (22 to 35)	32 (10 to 55)	+10%	3 (-13 to 21)

HI burden is expressed as the number of days lost per 1000 hours of exposure.

### Replacement of head coach

The head coach replacement was evenly distributed between the start and middle of the season. We could not find any significant increase in the HI burden by replacing the head coach alone before or during a new season (table 1).

### Replacement of head fitness/performance coach

The head fitness/performance coach replacement before or during the season was noted in 36% (15/42) of team seasons. Such changes were associated with an increase of 63% in HI burden compared with teams that did not have the position replaced. This increase in injury burden was significant when these alterations occurred either before or during the season (95% CI 3 to 28, p=0.017) (table 1).

### Replacements of head team doctor or head physiotherapist

Replacements of the main responsible team doctor or physiotherapist before or during a season were uncommon. Such changes occurred every sixth team season (7 changes during 42 team seasons,17%, see table 1). A change of head physiotherapist was only associated with a non-significant 10% increase in HI. Still, a change of team doctor was associated with an 81% increase in HI burden compared with teams that did not have the position replaced (95% CI 5 to 37, p=0.011).

### A new head coach bringing his own fitness/performance coach

As seen in table 2, most head coaches (70%) who started their role at the beginning of the season chose to introduce their own fitness/performance coach to the new club. When the head coach replacement occurred during the season, only four out of nine brought their own fitness coach. In five out of nine cases (one club did not answer Question 24), the fitness coach was brought in by the club itself. Such changes were associated with a considerable increase in HI burden compared with seasons where teams did not report significant replacements.

**Table 2:** Hamstring injury (HI) burden for seasons where a new coach brought their own fitness/performance coach compared with team seasons where the club brought in the head of fitness/performance coach

New head coach bringing own fitness/performance coach	Frequency	HI burden for team seasons where a new head coach brought in their own fitness/performance coach (95% CI)	HI burden for team seasons where the club brought in the fitness/performance coach (95% CI)	Change of HI burden	HI burden difference (head coach brought in – club brought in) (95% CI)
New head coach at the start of the season bringing own fitness/performance coach	7/10=70%	44 (32 to 54)	16 (12 to 19)	+175%	**28 (7 to 32)**
New head coach during season bringing own fitness/performance coach	4/9=44%^*^	48 (14 to 83)	16 (8 to 25)	+300%	32 (−17 to 81)†
New head coach before or during the season bringing own fitness/performance coach	11/19=58%^*^	45 (34 to 60)	16 (12 to 22)	+276%	**29 (14 to 45)†**

HI burden is expressed as the number of days lost per 1000 hours of exposure.

Bold CIs are significant at 0.05 level.

*One club did not provide an answer to Question 24 (Was the performance/fitness coach of the 2021/2022 season brought in by the head coach or by the club?).

†Equal variance assumed (SD_higher_/SD_lower_<2, except for where equal variance not assumed were used.

A new head coach bringing his own fitness/performance coach was associated with up to a three times increase in HI burden (table 2). The substitution of the head coach at the start or during the season, with the introduction of their own fitness coach/performance coach, was highly associated with an increase in HI burden: 276% (95% CI 14 to 45, p<0.001).

## Discussion

The principal finding of the current study was that changes in the main staff around a team were often correlated to an increase in HI burden. The most dramatic change in HI burden occurred when a new coach introduced their own fitness/performance coach to the new club. Furthermore, changes within the medical team, such as a change of team doctor, also seem to be associated with an increase in the HI burden.

### Can we explain why a change of head coach is common in male elite-level football teams?

The purpose of professional football teams is to win games and trophies. A high-performance soccer coach’s ability and reputation are based on match wins and losses. Consequently, a low win–loss ratio could trigger a coaching change at any stage of the season.^16 17^

The replacement of the head coach is frequent in elite football, but its impact on team success is debatable.^12 16–18^ A recent study by Goméz *et al*^16^ demonstrated that introducing a new coach significantly improved the team’s short-term performance. However, this positive impact declined in the longer term (>10 matches). Head coaches at the elite level are required to deal with several issues that may affect the team’s performance over the season, such as player injuries, congested match fixtures, player recruitment, daily practice, match tactical preparation and media.^16 17^ Among the board members of football teams and fans, there is a common belief that replacing the manager can improve the clubs’ results.^6^

### Can we explain why a new head coach for a season was associated with a lower increase in HI burden compared with a change of fitness/performance coach and team doctor?

In elite football, the head coach is much like an orchestra conductor.^19^ The modern manager has to show diplomatic communication skills and needs to be able to delegate effectively among the backroom team.^19^ The head of fitness/performance determines the physical load of the training within the remit of the coaching philosophy.^19^

Since players’ loading and fatigue are associated with HI,^7^ the fitness/performance coach might be more influential on HI than the manager, who remains the leader of the whole orchestra. The team doctor may influence the planning of physical load with the rest of the medical team but is also responsible for treating injuries and part of the discussion of return to play.

### Can we explain why a fitness/performance coach change was associated with an increase in HI burden?

The fitness coach is part of the coaching staff and the main person responsible for the player’s physical performance. This role is not only about ensuring that each player is performing to their full physical capacity but, more importantly, about developing a physical performance plan that is fully aligned with the coaching philosophy of the head coach.^20^

Many fitness/performance coaches have opinions on training content and periodisation.^21^ As a result, any new staff member could impose a different training philosophy on the new team, with the risk of significantly modifying players’ training demands and periodisation. This alteration may result in a higher HI burden. Overloading or underloading the players during training sessions might cause them to be under-prepared for the matches’ demands, increasing their risk of injuries, especially muscle injuries. Furthermore, when a fitness/performance coach starts to work for a new club, they enter a different subculture of people who are used to unique football references, terminology and language disparities, which may become a problem.^21^ To conclude, with the entrance of a new fitness coach, players might have to learn different football terminology to understand the coach’s messages and directions.^21^

It could be speculated whether there might also be a mental aspect that leads all players (including the ‘substitutes’) to train harder to ‘show’ themselves to the new physical coach. This might play a role in the rise of the HI burden.

### Can we explain why a mid-season head coach turnover does not influence HI’s burden?

We have shown the importance of thorough season preparation.^22^ This has already been performed when a head coach is fired during an ongoing season.

Further, it was common for head coaches to bring their own fitness/performance coach to a new club, and it was also frequent that the fitness/performance coach left the club together with the head coach. When replacements occur during a season, there might be an opening position for a new fitness/performance coach. In the present study, most teams (five out of nine) brought in a new head of fitness/performance from within the club. This results in a low HI burden for the season compared with the four teams where a new head coach introduced his own fitness/performance coach during the season (HI burden 16 vs 48 = +300%). A reasonable explanation for this finding would be that a fitness/performance coach selected from within the club would already know the players, be aware of the club’s performance philosophy and have established communication, including an agreed football language to use with players.

### Can we explain why a change of head team doctor is rare but associated with an increasing HI burden?

The substitution of the main medical doctor may increase the HI injury burden. However, a change in the medical team is rare. As previously shown in the ECIS, internal communication within a team is associated with both injury rates and player availability.^23^ The communication between the head coach and the medical team is the most influential.^23^

### Establishing good communication and a trustful relationship takes time

The main team doctor has a central role in the communication of medical information towards the head coach. When there are changes in the medical staff, it takes time to establish a trusting relationship.

For this reason, achieving effective communication can be challenging, which may explain the increase of the HI burden when a new medical team member is introduced.

Injury prevention strategies should be highly individualised at an elite level, and individualised care requires well-developed communication between staff members.^23^

Communication among staff is not the only crucial factor. Also, a new team doctor needs time to familiarise themselves with the players, and this process may influence injury rates.

### What does this mean practically?

The head of performance/fitness coach on load management and the head of the medical staff on medical information fulfil crucial roles in the communication within the expanding staff of professional football.

Any role replacement will cause a change in communication, which is essential in this environment. Changes might need to be made with care, and extra time should be invested to optimise communication and establish a trusting relationship between the head coach, head of the medical staff and head of performance/fitness coach.

The transition of the head, the performance/fitness coach and the medical team must be done with the utmost care and a thorough and detailed handover with the former staff. However, this is not always easy since communication between new and former staff may be less effective in most cases.

Universal football terminology and language are desirable to improve communication and avoid misunderstandings. Take aviation, for example, where pilots and air traffic controllers worldwide use the same established terminology and act similarly.^21^ The difference between a football action like pressing (an action involving communication, decision-making and execution) and a basic action like running (no interaction with the environment) is an example.^21^

This study indicates that clubs should have a stable structure, even if the head coach changes. Assuring coaching staff consistency might contribute to reducing the number of HIs. Increased trainers’ and medical teams’ attentiveness and proactivity during coaching transitions could be key. Caution on the impact of the new training regimens might be needed to decrease hamstring injury risk in professional football.^16^

Fitness coaches could be advised to use a ‘minimal intervention’ strategy during the mid-season and introduce new exercises and training gradually to avoid a boomerang effect.

### Methodological considerations

A main strength of this study is that its design follows the international consensus statements and reporting guidelines for epidemiological research in sports.^13–15^ The hamstring burden data were collected prospectively and should be considered robust since the ECIS has been ongoing for 21 years. It is an appropriate, reliable and useful tool for evaluating injury risk and injury patterns in elite male footballers. Furthermore, all 14 teams answered the questionnaire, thus a response rate of 100%.

The study, however, has its limitations. The study is limited by a relatively small sample size and a short observation period. Even in this relatively small sample size, a fitness/performance coach or team doctor change was associated with a significant increase in HI burden compared with seasons without such a change, indicating a robust finding and a strong relation.

However, the risk of recall bias should be considered since the change of staff data was retrospectively collected for the three latest seasons. It is reasonable that changes in coaching staff can be reliably recorded, even retrospectively. The main responsible team doctor provided the change of staff data in the 14 teams, and 8 of them were in this position for all three seasons and were aware of changes in the head of departments. Doctors at this level work with their teams full-time and see other staff members daily. As a result of their medical training, they are, in our opinion, in the best position to deliver reliable data on changes of head positions in staff around the team.

Further, we also checked the change of head coach in the teams by Transfermarkt.

Finally, as this is a descriptive study, we cannot infer any causality between the change of staff around a team and HI rates. However, despite not being causal, the new information and the shown associations should still have practical implications.

## Conclusion

Transferring their own fitness/performance coaches is common for managers entering a new elite male football club. Findings from this paper indicate that this trend could lead to up to three times increase in HI burden. Replacing the team doctor, which is less common, was also associated with an obvious increase in HI burden. If you replace your head coach, you should be recommended to keep other parts of the team intact.

## Data Availability

No data are available.
