# Supplementary material for: Changes in head staff members in male elite-level football teams are associated with increased hamstring injury burden for that season: the UEFA Elite Club Injury Study
Source: BMJ Open Sport Exerc Med. 2023 Nov 15;9(4):e001640. doi: 10.1136/bmjsem-2023-001640 (PMC10660205; doi:10.1136/bmjsem-2023-001640)
Supplement: Supplementary data [file bmjsem-2023-001640supp001.pdf]

Box 1. Survey questions

1. Please, enter the Code number of your club or your initials.
2. Did you have a change of head coach before the 2019/20 season (= a new coach for the season)?
3. Did you have a change of head coach during the 2019/20 season?
4. If answered Yes at question 3: in which month did the change of head coach occur?
5. Did you have a change of performance/fitness coach before the 2019/20 season (= a new performance/fitness coach for the season)?
6. Was the performance/fitness coach of the 2019/20 season brought in by the head coach or the club?
7. Did you have a change of performance/fitness coach during the 2019/20 season?
8. If answered yes at question 7, was the new performance/fitness coach during the ongoing 2019/20 season brought in by the head coach or by the club?
9. Did you have a change of the main responsible team doctor before the 2019/20 season (= a new main responsible team doctor for the season)?
10. Did you have a change of the head physiotherapist before the 2019/20 season (= a new head physiotherapist for the season)?
11. Did you have a change of head coach before the 2020/21 season (= a new coach for the season)?
12. Did you have a change of head coach during the 2020/21 season?
13. If answered Yes at question 12: in which month did the change of head coach occur?
14. Did you have a change of performance/fitness coach before the 2020/21 season (= a new performance/fitness coach for the season)?
15. Was the performance/fitness coach of the 2020/21 season brought in by the head coach or by the club?
16. Did you have a change of performance/fitness coach during the 2020/21 season?
17. If answered yes at question 16, was the new performance/fitness coach during the ongoing 2020/21 season brought in by the head coach or by the club?
18. Did you have a change of the main responsible team doctor before the 2020/21 season (= a new main responsible team doctor for the season)?

19. Did you have a change of the head physiotherapist before the 2020/21 season (= a new head physiotherapist for the season)?
20. Did you have a change of head coach before the 2021/22 season (= a new coach for the season)?
21. Did you have a change of head coach during the 2021/22 season?
22. If answered Yes at question 21: in which month did the change of head coach occur?
23. Did you have a change of performance/fitness coach before the 2021/22 season (= a new performance/fitness coach for the season)?
24. Was the performance/fitness coach of the 2021/22 season brought in by the head coach or by the club?
25. Did you have a change of performance/fitness coach during the 2021/22 season?
26. If answered yes at question 25, was the new performance/fitness coach during the ongoing 2021/22 season brought in by the head coach or by the club?
27. Did you have a change of the main responsible team doctor before the 2021/22 season (= a new main responsible team doctor for the season)?
28. Did you have a change of the head physiotherapist before the 2021/22 season (= a new head physiotherapist for the season)?
